# Supplementary material for: Long jumpers with and without a transtibial amputation have different three-dimensional centre of mass and joint take-off step kinematics
Source: R Soc Open Sci. 2019 Apr 17;6(4):190107. doi: 10.1098/rsos.190107 (PMC6502388; doi:10.1098/rsos.190107)
Supplement: Peak joint angles. [file rsos190107supp1.pdf]

Supplementary Table 1, for Funken et al. (2019) Royal Society Open Science, 6:190107.

Supplementary Table 1. Peak joint angles.

	Athletes with BKA			Non-amputee athletes			Difference BKA to nonAMP	
**Peak joint angles [°]**	Mean (SD)	Min	Max	Mean (SD)	Min	Max	[%]	p
Ankle[a]								
Dorsiflexion (+)	27.9 (3.7)	24.4	31.8	17.6 (4.1)	13.4	23.6	58.8	**.017**
Plantarflexion (-)	-1.1 (5.2)	4.7	-5.6	-31.5 (5.5)	-21.0	-38.1	-96.5	**.017**
Eversion (+)	2.8 (3.9)	-1.22	6.6	15.8 (5.4)	6.3	22.4	-82.6	**.033**
Inversion (-)	0.9 (4.1)	4.7	-3.5	-4.8 (4.2)	1.54	-9.9	-118.7	.117
External rotation (+)	5.2 (2.8)	3.1	8.4	-1.2 (2.2)	-3.8	2.4	-529.0	**.017**
Internal rotation (-)	1.2 (4.9)	6.6	-3.0	-11.8 (1.8)	-8.7	-14.5	-110.7	**.017**
Knee								
Flexion (+)	28.7 (0.5)	28.3	29.3	48.2 (5.3)	41.0	55.0	-40.3	**.017**
Extension (-)	6.2 (4.3)	10.9	2.6	-3.8 (6.0)	11.5	-4.1	64.8	.517
Abduction (+)	4.9 (3.9)	2.2	9.3	9.4 (4.5)	3.3	16.2	-48.3	.117
Adduction (-)	-1.1 (3.4)	2.4	-4.2	-1.8 (3.1)	2.2	-5.9	-37.0	.833
External rotation (+)	3.8 (2.7)	0.8	6.2	6.8 (2.9)	2.0	10.3	-44.2	.183
Internal rotation (-)	0.2 (1.4)	1.6	-1.2	-10.8 (3.1)	-6.4	-14.3	-101.5	**.017**
Hip								
Flexion (+)	16.6 (0.8)	15.9	17.4	34.4 (5.1)	27.2	42.2	-51.8	**.017**
Extension (-)	-19.1 (5.9)	-13.8	-25.4	-25.7 (3.4)	-19.9	-31.3	-25.7	.117
Abduction (+)	11.3 (3.7)	8.8	15.6	18.2 (3.3)	15.2	24.6	-37.8	**.033**
Adduction (-)	-3.7 (7.7)	5.1	-9.2	-8.2 (5.8)	-2.2	-15.7	-55.0	.667
External rotation (+)	5.5 (16.5)	-6.8	24.3	24.0 (8.3)	11.5	37.2	-77.0	.183
Internal rotation (-)	-11.0 (13.0)	3.2	-22.3	-1.3 (8.3)	8.0	-12.9	748.8	.183

Peak joint angles as mean with standard deviations (SD), within-group minimum and maximum values for athletes with a below the knee amputation (BKA) and non-amputee athletes (nonAMP) during the take-off step of the long jump. The differences between mean values from non-amputee athletes and athletes with BKA are presented as percentages. Bold p-values indicate significant differences.

[a] Ankle in the BKA group refers to the point of the prosthesis' greatest curvature defined as the prosthetic ankle joint
